# 5-Bromo-2-[5-(4-nitro­phen­yl)-3-phenyl-4,5-dihydro-1*H*-pyrazol-1-yl]pyrimidine

**DOI:** 10.1107/S1600536809048600

**Published:** 2009-11-21

**Authors:** Jia Hao Goh, Hoong-Kun Fun, Adithya Adhikari, B. Kalluraya

**Affiliations:** a X-ray Crystallography Unit, School of Physics, Universiti Sains Malaysia, 11800 USM, Penang, Malaysia; b Department of Studies in Chemistry, Mangalore University, Mangalagangotri, Mangalore 574 199, India

## Abstract

In the title pyrazoline compound, C_19_H_14_BrN_5_O_2_, the essentially planar pyrazoline and pyrimidine rings [maximum deviations = 0.013 (1) and 0.009 (1) Å, respectively] are inclined slightly to one another, making a dihedral angle of 10.81 (10)°. The nitro­benzene unit is almost perpendicular to the attached pyrazoline ring, as indicated by the dihedral angle of 84.61 (8)°. In the crystal structure, inter­molecular C—H⋯N contacts link the mol­ecules into dimers in an anti­parallel manner. These dimers are further linked into one-dimensional chains along the *b* axis *via* C—H⋯O contacts. The crystal structure is consolidated by three different inter­molecular π–π inter­actions [range of centroid–centroid distances = 3.5160 (11)–3.6912 (11) Å].

## Related literature

For general background to and applications of the title compound, see: Hegde *et al.* (2006 ▶); Kalluraya & Chimbalkar (2001 ▶); Kalluraya *et al.* (2001 ▶); Rai *et al.* (2008 ▶); Rathish *et al.* (2009 ▶); Tawab *et al.* (1960 ▶). For closely related structures, see: Goh *et al.* (2009**a* ▶,b* ▶). For the stability of the temperature controller used for the data collection, see: Cosier & Glazer (1986 ▶).
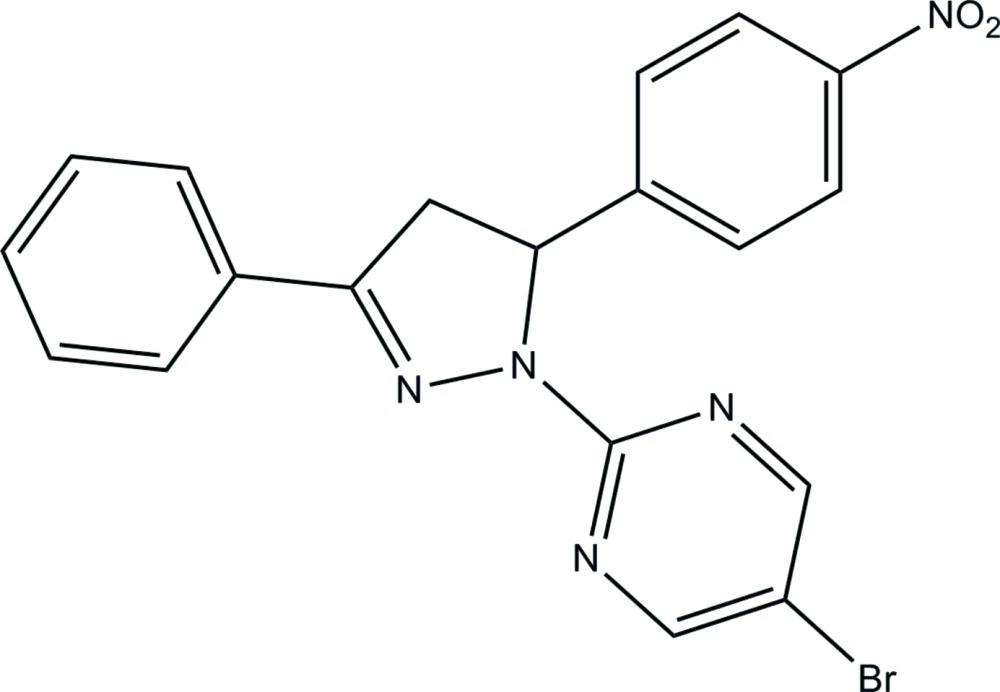


## Experimental

### 

#### Crystal data

C_19_H_14_BrN_5_O_2_
*M* *_r_* = 424.26
Triclinic, 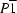

*a* = 6.9709 (1) Å
*b* = 11.6500 (2) Å
*c* = 12.4365 (2) Å
α = 114.969 (1)°
β = 103.303 (1)°
γ = 91.560 (1)°
*V* = 882.12 (2) Å^3^
*Z* = 2
Mo *K*α radiation
μ = 2.36 mm^−1^
*T* = 100 K
0.33 × 0.22 × 0.12 mm

#### Data collection

Bruker SMART APEXII CCD area-detector diffractometer
Absorption correction: multi-scan (**SADABS**; Bruker, 2005 ▶) *T*_min_ = 0.510, *T*_max_ = 0.760
28197 measured reflections
3804 independent reflections
3431 reflections with *I* > 2σ(*I*)
*R*_int_ = 0.026

#### Refinement

*R*[*F* ^2^ > 2σ(*F* ^2^)] = 0.024
*wR*(*F* ^2^) = 0.060
*S* = 1.05
3804 reflections
300 parameters
All H-atom parameters refined
Δρ_max_ = 0.49 e Å^−3^
Δρ_min_ = −0.30 e Å^−3^

### 

Data collection: *APEX2* (Bruker, 2005 ▶); cell refinement: *SAINT* (Bruker, 2005 ▶); data reduction: *SAINT*; program(s) used to solve structure: *SHELXTL* (Sheldrick, 2008 ▶); program(s) used to refine structure: *SHELXTL*; molecular graphics: *SHELXTL*; software used to prepare material for publication: *SHELXTL* and *PLATON* (Spek, 2009 ▶).

## Supplementary Material

Crystal structure: contains datablocks global, I. DOI: 10.1107/S1600536809048600/tk2577sup1.cif

Structure factors: contains datablocks I. DOI: 10.1107/S1600536809048600/tk2577Isup2.hkl

Additional supplementary materials:  crystallographic information; 3D view; checkCIF report

## Figures and Tables

**Table 1:** Hydrogen-bond geometry (Å, °)

*D*—H⋯*A*	*D*—H	H⋯*A*	*D*⋯*A*	*D*—H⋯*A*
C8—H8*B*⋯O2^i^	0.95 (2)	2.41 (2)	3.352 (2)	176.0 (17)
C11—H11*A*⋯N4^ii^	0.92 (2)	2.56 (2)	3.431 (2)	160.5 (18)
C19—H19*A*⋯O2^iii^	0.98 (2)	2.58 (2)	3.412 (3)	143.3 (17)

Symmetry codes: (i) 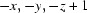; (ii) 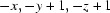; (iii) 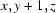.

**Fig. 1.**
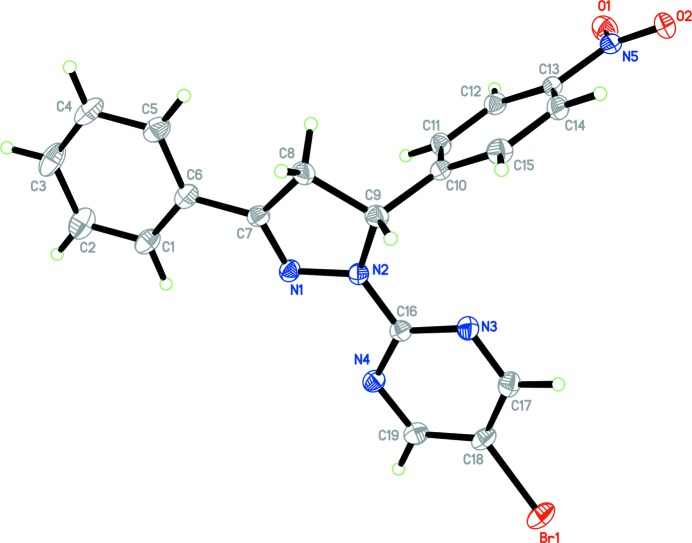
The molecular structure of (I), showing 50% probability displacement ellipsoids for non-H atoms and the atom-numbering scheme.

**Fig. 2.**
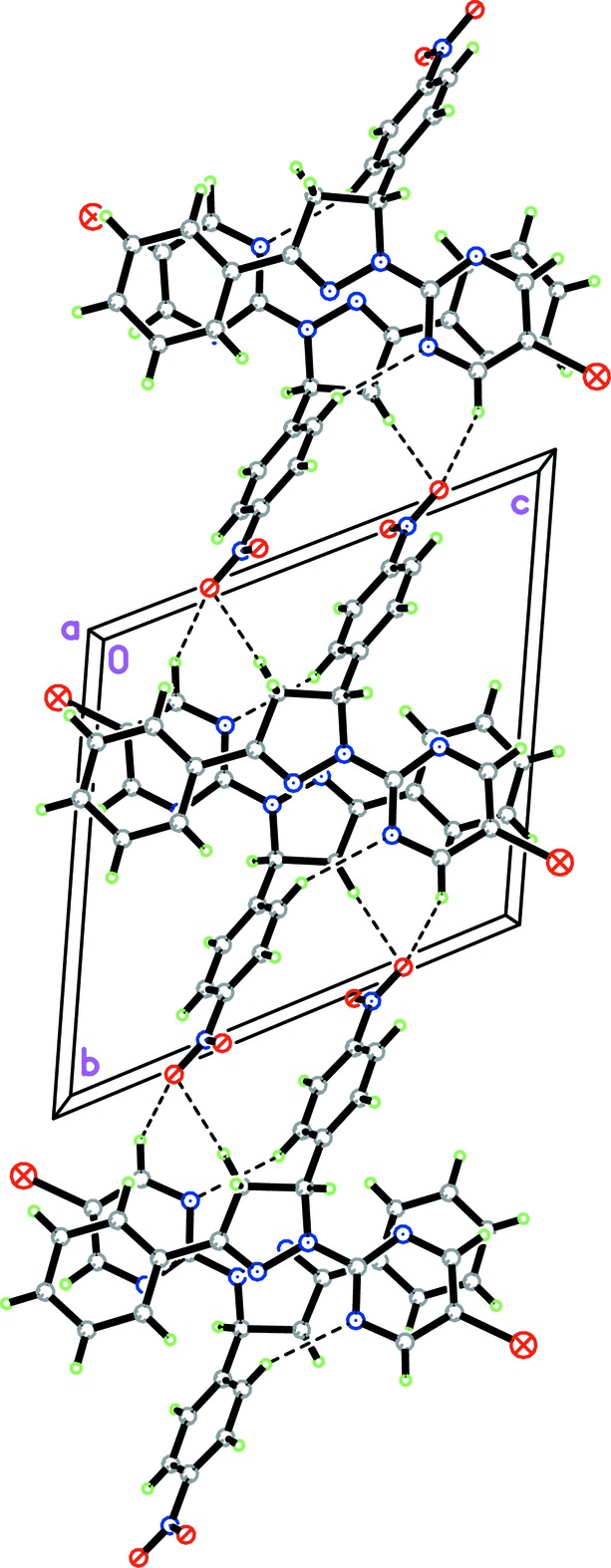
Part of the crystal structure of (I), viewed along the a axis, showing the dimers being linked into a 1-D chain along the b axis. Intermolecular contacts are shown as dashed lines.

## supplementary crystallographic information

### Comment

Pyrazoles and pyrazoline derivatives are an important class of heterocyclic compounds (Rai *et al.*, 2008; Hegde *et al.*, 2006). The addition of aliphatic diazo compounds to olefins leads to pyrazolines. Also, the addition of hydrazine or its derivatives to α, β-unsaturated aldehydes or ketones yields pyrazoline (Kalluraya & Chimbalkar, 2001). Pyrazoline derivatives have been found to possess potential anti-pyretic, analgesic (Tawab *et al.*, 1960), anti-inflammatory (Rathish *et al.*, 2009), and anti-microbial (Kalluraya *et al.*, 2001) properties. In the present work, an X-ray crystal structure analysis has been undertaken in order to determine the 3D chemical structure and also the crystal packing of the molecules. We herein report the synthesis and crystal structure of the title compound, (I).

In (I), Fig. 1, the pyrazoline (C7-C9/N1/N2) and pyrimidine (C16-C19/N3/N4) rings are essentially planar, with maximum deviations of 0.013 (1) and 0.009 (1) Å, respectively, for atoms N1 and C16. These two rings are slightly inclined to one another, making a dihedral angle of 10.81 (10)°. The nitrobenzene moiety is almost perpendicular to the attached pyrazoline ring, as indicated by the dihedral angle formed between the mean plane through C10-C15/N5/O1/O2 and the pyrazoline ring of 84.61 (8)°. The bond lengths and angles are consistent with those closely related structures (Goh *et al.*, 2009*a,b*).

In the crystal structure (Fig. 2), intermolecular C11—H11A···N4 contacts (Table 1) link the molecules into dimers in an anti-parallel manner. These dimers are further linked into a 1-D chain along the *b* axis by intermolecular C8—H8B···O2 and C19—H19A···O2 contacts (Table 1). The crystal structure is consolidated by three different weak π–π interactions involving the pyrazoline (*Cg*1), pyrimidine (*Cg*2) and C1-C6 benzene (*Cg*3) rings [*Cg*1···*Cg*1^iv^ = 3.5160 (11) Å, *Cg*2···*Cg*3^ii^ = 3.6912 (11) Å and *Cg*2···*Cg*3^iv^ = 3.5779 (11) Å, respectively; (ii) -x, 1-y, 1-z and (iv) 1-x, 1-y, 1-z].

### Experimental

A mixture of 5-bromo-2-hydrazinopyrimidine (0.01 mol) and 3-(4-nitrophenyl)-1-phenyl-prop-2-en-1-one (0.01 mol) was taken in acetic acid (20 ml), and two drops of concentrated H_2_SO_4_ added. The mixture was refluxed for 4 h. The precipitated solids were filtered, dried and recrystallized from ethanol. The single crystals were obtained from a mixture of ethanol and DMF by slow evaporation.

### Refinement

All the H atoms were located from difference Fourier map [range of C—H = 0.91 (2) – 0.995 (19) Å] and allowed to refine freely. The reflections (001) and (011) were omitted as the intensities were affected by the beam-stop.

### Crystal data

**Table:**

C_19_H_14_BrN_5_O_2_	*Z* = 2
*M**_r_* = 424.26	*F*(000) = 428
Triclinic, *P*1	*D*_x_ = 1.597 Mg m^−^^3^
Hall symbol: -P 1	Mo *K*α radiation, λ = 0.71073 Å
*a* = 6.9709 (1) Å	Cell parameters from 9873 reflections
*b* = 11.6500 (2) Å	θ = 3.0–33.9°
*c* = 12.4365 (2) Å	µ = 2.36 mm^−^^1^
α = 114.969 (1)°	*T* = 100 K
β = 103.303 (1)°	Block, green
γ = 91.560 (1)°	0.33 × 0.22 × 0.12 mm
*V* = 882.12 (2) Å^3^	

### Data collection

**Table:**

Bruker SMART APEXII CCD area-detector diffractometer	3804 independent reflections
Radiation source: fine-focus sealed tube	3431 reflections with *I* > 2σ(*I*)
graphite	*R*_int_ = 0.026
φ and ω scans	θ_max_ = 27.0°, θ_min_ = 2.0°
Absorption correction: multi-scan (*SADABS*; Bruker, 2005)	*h* = −8→8
*T*_min_ = 0.510, *T*_max_ = 0.760	*k* = −14→14
28197 measured reflections	*l* = −15→15

### Refinement

**Table:**

Refinement on *F*^2^	Primary atom site location: structure-invariant direct methods
Least-squares matrix: full	Secondary atom site location: difference Fourier map
*R*[*F*^2^ > 2σ(*F*^2^)] = 0.024	Hydrogen site location: inferred from neighbouring sites
*wR*(*F*^2^) = 0.060	All H-atom parameters refined
*S* = 1.05	*w* = 1/[σ^2^(*F*_o_^2^) + (0.0288*P*)^2^ + 0.5594*P*] where *P* = (*F*_o_^2^ + 2*F*_c_^2^)/3
3804 reflections	(Δ/σ)_max_ = 0.001
300 parameters	Δρ_max_ = 0.49 e Å^−^^3^
0 restraints	Δρ_min_ = −0.30 e Å^−^^3^

### Special details

**Table:**

Experimental. The crystal was placed in the cold stream of an Oxford Cyrosystems Cobra open-flow nitrogen cryostat (Cosier & Glazer, 1986) operating at 100.0 (1)K.
Geometry. All esds (except the esd in the dihedral angle between two l.s. planes) are estimated using the full covariance matrix. The cell esds are taken into account individually in the estimation of esds in distances, angles and torsion angles; correlations between esds in cell parameters are only used when they are defined by crystal symmetry. An approximate (isotropic) treatment of cell esds is used for estimating esds involving l.s. planes.
Refinement. Refinement of F^2^ against ALL reflections. The weighted R-factor wR and goodness of fit S are based on F^2^, conventional R-factors R are based on F, with F set to zero for negative F^2^. The threshold expression of F^2^ > 2sigma(F^2^) is used only for calculating R-factors(gt) etc. and is not relevant to the choice of reflections for refinement. R-factors based on F^2^ are statistically about twice as large as those based on F, and R- factors based on ALL data will be even larger.

### Fractional atomic coordinates and isotropic or equivalent isotropic displacement parameters (Å^2^)

**Table:**

	*x*	*y*	*z*	*U*_iso_*/*U*_eq_	
Br1	0.32778 (3)	0.891599 (17)	1.068122 (15)	0.02770 (7)	
O1	−0.3115 (2)	0.02211 (12)	0.64236 (12)	0.0274 (3)	
O2	−0.0404 (2)	−0.01117 (12)	0.74511 (11)	0.0258 (3)	
N1	0.2287 (2)	0.49488 (13)	0.46957 (12)	0.0175 (3)	
N2	0.2681 (2)	0.47661 (13)	0.57546 (12)	0.0177 (3)	
N3	0.3203 (2)	0.54232 (13)	0.78176 (13)	0.0191 (3)	
N4	0.2364 (2)	0.68890 (13)	0.69321 (13)	0.0182 (3)	
N5	−0.1294 (2)	0.03714 (13)	0.68034 (13)	0.0205 (3)	
C1	0.1859 (3)	0.48220 (17)	0.22862 (16)	0.0224 (4)	
C2	0.1604 (3)	0.4683 (2)	0.10980 (17)	0.0282 (4)	
C3	0.1682 (3)	0.3501 (2)	0.01564 (17)	0.0300 (4)	
C4	0.2012 (3)	0.24641 (19)	0.04084 (17)	0.0275 (4)	
C5	0.2323 (3)	0.26060 (18)	0.16071 (16)	0.0236 (4)	
C6	0.2252 (2)	0.37903 (16)	0.25600 (15)	0.0201 (3)	
C7	0.2607 (2)	0.39338 (15)	0.38231 (15)	0.0176 (3)	
C8	0.3349 (3)	0.29328 (16)	0.42148 (17)	0.0228 (4)	
C9	0.3406 (3)	0.35347 (16)	0.55889 (15)	0.0191 (3)	
C10	0.2129 (3)	0.27153 (15)	0.59130 (14)	0.0175 (3)	
C11	0.0070 (3)	0.24832 (16)	0.54497 (15)	0.0188 (3)	
C12	−0.1072 (3)	0.17094 (16)	0.57339 (15)	0.0191 (3)	
C13	−0.0101 (3)	0.11782 (15)	0.64817 (15)	0.0178 (3)	
C14	0.1942 (3)	0.13677 (16)	0.69325 (16)	0.0211 (3)	
C15	0.3060 (3)	0.21486 (16)	0.66433 (16)	0.0213 (3)	
C16	0.2747 (2)	0.57397 (15)	0.68747 (15)	0.0165 (3)	
C17	0.3326 (3)	0.63670 (17)	0.89244 (16)	0.0207 (3)	
C18	0.2999 (3)	0.75881 (16)	0.90934 (15)	0.0204 (3)	
C19	0.2496 (3)	0.78065 (16)	0.80562 (16)	0.0201 (3)	
H1A	0.177 (3)	0.563 (2)	0.2909 (19)	0.025 (5)*	
H2A	0.138 (4)	0.540 (2)	0.092 (2)	0.038 (6)*	
H3A	0.154 (3)	0.340 (2)	−0.066 (2)	0.037 (6)*	
H4A	0.206 (3)	0.169 (2)	−0.020 (2)	0.024 (5)*	
H5A	0.257 (3)	0.190 (2)	0.178 (2)	0.028 (5)*	
H8A	0.467 (3)	0.2771 (19)	0.4110 (19)	0.024 (5)*	
H8B	0.250 (3)	0.214 (2)	0.378 (2)	0.031 (6)*	
H9A	0.478 (3)	0.3691 (17)	0.6123 (17)	0.013 (4)*	
H11A	−0.055 (3)	0.2853 (18)	0.4970 (18)	0.019 (5)*	
H12A	−0.245 (3)	0.1549 (19)	0.5429 (19)	0.024 (5)*	
H14A	0.258 (3)	0.0991 (19)	0.7416 (19)	0.025 (5)*	
H15A	0.446 (3)	0.2291 (19)	0.6952 (19)	0.025 (5)*	
H17A	0.372 (3)	0.6149 (19)	0.9598 (19)	0.022 (5)*	
H19A	0.224 (3)	0.8658 (19)	0.8141 (18)	0.019 (5)*	

### Atomic displacement parameters (Å^2^)

**Table:**

	*U*^11^	*U*^22^	*U*^33^	*U*^12^	*U*^13^	*U*^23^
Br1	0.02884 (11)	0.02882 (11)	0.01659 (9)	0.00509 (7)	0.00679 (7)	0.00134 (7)
O1	0.0263 (7)	0.0275 (7)	0.0323 (7)	0.0020 (5)	0.0112 (6)	0.0154 (6)
O2	0.0386 (8)	0.0213 (6)	0.0223 (6)	0.0044 (5)	0.0086 (6)	0.0139 (5)
N1	0.0171 (7)	0.0197 (7)	0.0145 (6)	0.0012 (5)	0.0029 (5)	0.0070 (5)
N2	0.0231 (8)	0.0155 (6)	0.0160 (7)	0.0037 (5)	0.0070 (6)	0.0073 (5)
N3	0.0199 (7)	0.0202 (7)	0.0180 (7)	0.0023 (6)	0.0060 (6)	0.0087 (6)
N4	0.0185 (7)	0.0179 (7)	0.0174 (7)	0.0043 (5)	0.0047 (6)	0.0068 (6)
N5	0.0302 (9)	0.0147 (6)	0.0173 (7)	0.0036 (6)	0.0107 (6)	0.0055 (6)
C1	0.0190 (9)	0.0260 (9)	0.0173 (8)	0.0058 (7)	0.0023 (7)	0.0061 (7)
C2	0.0244 (10)	0.0390 (11)	0.0209 (9)	0.0122 (8)	0.0031 (7)	0.0140 (8)
C3	0.0235 (10)	0.0459 (12)	0.0152 (8)	0.0112 (8)	0.0028 (7)	0.0091 (8)
C4	0.0202 (9)	0.0321 (10)	0.0173 (8)	0.0036 (8)	0.0049 (7)	−0.0012 (8)
C5	0.0185 (9)	0.0247 (9)	0.0219 (9)	0.0023 (7)	0.0063 (7)	0.0045 (7)
C6	0.0137 (8)	0.0250 (9)	0.0169 (8)	0.0010 (6)	0.0040 (6)	0.0049 (7)
C7	0.0149 (8)	0.0172 (7)	0.0179 (8)	−0.0004 (6)	0.0050 (6)	0.0051 (6)
C8	0.0315 (10)	0.0164 (8)	0.0246 (9)	0.0043 (7)	0.0156 (8)	0.0086 (7)
C9	0.0212 (9)	0.0161 (7)	0.0209 (8)	0.0036 (6)	0.0081 (7)	0.0078 (6)
C10	0.0236 (9)	0.0140 (7)	0.0149 (7)	0.0033 (6)	0.0077 (6)	0.0050 (6)
C11	0.0225 (9)	0.0184 (8)	0.0179 (8)	0.0055 (7)	0.0056 (7)	0.0099 (7)
C12	0.0204 (9)	0.0187 (8)	0.0183 (8)	0.0046 (7)	0.0060 (7)	0.0075 (7)
C13	0.0252 (9)	0.0137 (7)	0.0153 (7)	0.0028 (6)	0.0085 (7)	0.0057 (6)
C14	0.0278 (10)	0.0197 (8)	0.0179 (8)	0.0066 (7)	0.0054 (7)	0.0103 (7)
C15	0.0194 (9)	0.0216 (8)	0.0212 (8)	0.0032 (7)	0.0030 (7)	0.0092 (7)
C16	0.0136 (8)	0.0174 (8)	0.0173 (8)	0.0013 (6)	0.0048 (6)	0.0064 (6)
C17	0.0191 (9)	0.0255 (9)	0.0180 (8)	0.0028 (7)	0.0059 (7)	0.0095 (7)
C18	0.0176 (8)	0.0222 (8)	0.0152 (8)	0.0020 (7)	0.0049 (6)	0.0023 (7)
C19	0.0187 (9)	0.0192 (8)	0.0204 (8)	0.0046 (7)	0.0051 (7)	0.0067 (7)

### Geometric parameters (Å, °)

**Table:**

Br1—C18	1.8863 (16)	C5—H5A	0.94 (2)
O1—N5	1.228 (2)	C6—C7	1.468 (2)
O2—N5	1.2337 (19)	C7—C8	1.504 (2)
N1—C7	1.292 (2)	C8—C9	1.539 (2)
N1—N2	1.3886 (19)	C8—H8A	0.97 (2)
N2—C16	1.365 (2)	C8—H8B	0.95 (2)
N2—C9	1.481 (2)	C9—C10	1.519 (2)
N3—C17	1.332 (2)	C9—H9A	0.995 (19)
N3—C16	1.346 (2)	C10—C11	1.391 (2)
N4—C19	1.334 (2)	C10—C15	1.393 (2)
N4—C16	1.348 (2)	C11—C12	1.389 (2)
N5—C13	1.471 (2)	C11—H11A	0.91 (2)
C1—C2	1.384 (3)	C12—C13	1.387 (2)
C1—C6	1.399 (3)	C12—H12A	0.93 (2)
C1—H1A	0.95 (2)	C13—C14	1.379 (3)
C2—C3	1.394 (3)	C14—C15	1.390 (2)
C2—H2A	0.96 (2)	C14—H14A	0.93 (2)
C3—C4	1.383 (3)	C15—H15A	0.94 (2)
C3—H3A	0.95 (2)	C17—C18	1.381 (3)
C4—C5	1.392 (3)	C17—H17A	0.96 (2)
C4—H4A	0.91 (2)	C18—C19	1.388 (2)
C5—C6	1.400 (2)	C19—H19A	0.98 (2)
			
C7—N1—N2	107.71 (14)	N2—C9—C10	113.23 (14)
C16—N2—N1	121.50 (13)	N2—C9—C8	101.32 (13)
C16—N2—C9	123.73 (14)	C10—C9—C8	113.11 (14)
N1—N2—C9	113.65 (13)	N2—C9—H9A	110.2 (10)
C17—N3—C16	115.56 (15)	C10—C9—H9A	107.2 (10)
C19—N4—C16	115.45 (14)	C8—C9—H9A	111.9 (10)
O1—N5—O2	123.49 (14)	C11—C10—C15	119.99 (15)
O1—N5—C13	118.57 (14)	C11—C10—C9	121.01 (15)
O2—N5—C13	117.94 (15)	C15—C10—C9	118.95 (15)
C2—C1—C6	120.44 (17)	C12—C11—C10	120.27 (16)
C2—C1—H1A	118.6 (12)	C12—C11—H11A	119.2 (12)
C6—C1—H1A	121.0 (12)	C10—C11—H11A	120.5 (12)
C1—C2—C3	120.15 (19)	C13—C12—C11	118.28 (16)
C1—C2—H2A	120.0 (14)	C13—C12—H12A	120.8 (12)
C3—C2—H2A	119.8 (14)	C11—C12—H12A	121.0 (12)
C4—C3—C2	119.95 (17)	C14—C13—C12	122.77 (15)
C4—C3—H3A	119.4 (14)	C14—C13—N5	118.41 (15)
C2—C3—H3A	120.6 (14)	C12—C13—N5	118.82 (15)
C3—C4—C5	120.16 (17)	C13—C14—C15	118.17 (16)
C3—C4—H4A	120.9 (13)	C13—C14—H14A	122.0 (13)
C5—C4—H4A	118.9 (13)	C15—C14—H14A	119.8 (13)
C4—C5—C6	120.28 (18)	C14—C15—C10	120.49 (17)
C4—C5—H5A	120.2 (13)	C14—C15—H15A	119.0 (12)
C6—C5—H5A	119.5 (13)	C10—C15—H15A	120.5 (12)
C1—C6—C5	118.96 (16)	N3—C16—N4	127.09 (15)
C1—C6—C7	121.19 (15)	N3—C16—N2	114.49 (14)
C5—C6—C7	119.85 (16)	N4—C16—N2	118.43 (14)
N1—C7—C6	121.79 (16)	N3—C17—C18	122.24 (16)
N1—C7—C8	114.40 (15)	N3—C17—H17A	115.3 (12)
C6—C7—C8	123.81 (15)	C18—C17—H17A	122.4 (12)
C7—C8—C9	102.87 (13)	C17—C18—C19	117.58 (15)
C7—C8—H8A	112.2 (12)	C17—C18—Br1	121.08 (13)
C9—C8—H8A	110.4 (12)	C19—C18—Br1	121.33 (13)
C7—C8—H8B	112.8 (13)	N4—C19—C18	122.05 (16)
C9—C8—H8B	111.1 (13)	N4—C19—H19A	118.1 (11)
H8A—C8—H8B	107.5 (18)	C18—C19—H19A	119.8 (11)
			
C7—N1—N2—C16	170.72 (14)	C15—C10—C11—C12	1.4 (2)
C7—N1—N2—C9	2.42 (18)	C9—C10—C11—C12	178.62 (15)
C6—C1—C2—C3	1.8 (3)	C10—C11—C12—C13	−0.2 (2)
C1—C2—C3—C4	0.1 (3)	C11—C12—C13—C14	−1.2 (2)
C2—C3—C4—C5	−1.8 (3)	C11—C12—C13—N5	179.18 (14)
C3—C4—C5—C6	1.6 (3)	O1—N5—C13—C14	178.59 (15)
C2—C1—C6—C5	−1.9 (3)	O2—N5—C13—C14	−0.9 (2)
C2—C1—C6—C7	177.49 (16)	O1—N5—C13—C12	−1.8 (2)
C4—C5—C6—C1	0.2 (3)	O2—N5—C13—C12	178.73 (14)
C4—C5—C6—C7	−179.20 (16)	C12—C13—C14—C15	1.5 (3)
N2—N1—C7—C6	177.49 (14)	N5—C13—C14—C15	−178.92 (15)
N2—N1—C7—C8	−2.15 (19)	C13—C14—C15—C10	−0.3 (3)
C1—C6—C7—N1	10.4 (2)	C11—C10—C15—C14	−1.1 (2)
C5—C6—C7—N1	−170.23 (16)	C9—C10—C15—C14	−178.41 (15)
C1—C6—C7—C8	−170.01 (16)	C17—N3—C16—N4	−1.6 (2)
C5—C6—C7—C8	9.4 (2)	C17—N3—C16—N2	178.34 (14)
N1—C7—C8—C9	1.11 (19)	C19—N4—C16—N3	1.4 (2)
C6—C7—C8—C9	−178.52 (15)	C19—N4—C16—N2	−178.54 (14)
C16—N2—C9—C10	68.9 (2)	N1—N2—C16—N3	−178.10 (14)
N1—N2—C9—C10	−123.07 (15)	C9—N2—C16—N3	−11.0 (2)
C16—N2—C9—C8	−169.64 (15)	N1—N2—C16—N4	1.8 (2)
N1—N2—C9—C8	−1.64 (17)	C9—N2—C16—N4	168.92 (14)
C7—C8—C9—N2	0.35 (16)	C16—N3—C17—C18	0.3 (2)
C7—C8—C9—C10	121.86 (15)	N3—C17—C18—C19	1.0 (3)
N2—C9—C10—C11	49.0 (2)	N3—C17—C18—Br1	−177.75 (12)
C8—C9—C10—C11	−65.5 (2)	C16—N4—C19—C18	0.1 (2)
N2—C9—C10—C15	−133.70 (16)	C17—C18—C19—N4	−1.2 (3)
C8—C9—C10—C15	111.76 (18)	Br1—C18—C19—N4	177.53 (13)

### Hydrogen-bond geometry (Å, °)

**Table:**

*D*—H···*A*	*D*—H	H···*A*	*D*···*A*	*D*—H···*A*
C8—H8B···O2^i^	0.95 (2)	2.41 (2)	3.352 (2)	176.0 (17)
C11—H11A···N4^ii^	0.92 (2)	2.56 (2)	3.431 (2)	160.5 (18)
C19—H19A···O2^iii^	0.98 (2)	2.58 (2)	3.412 (3)	143.3 (17)

Symmetry codes: (i) −*x*, −*y*, −*z*+1; (ii) −*x*, −*y*+1, −*z*+1; (iii) *x*, *y*+1, *z*.

## References

[bb1] Bruker (2005). *APEX2*, *SAINT* and *SADABS*. Bruker AXS Inc., Madison, Wisconsin, USA.

[bb2] Cosier, J. & Glazer, A. M. (1986). *J. Appl. Cryst.* **19**, 105–107.

[bb3] Goh, J. H., Fun, H.-K., Nithinchandra, & Kalluraya, B. (2009*a*). *Acta Cryst.* E**65**, o3088–o3089. 10.1107/S1600536809047217 PMC2972143 21578818

[bb4] Goh, J. H., Fun, H.-K., Nithinchandra, Rai, N. S. & Kalluraya, B. (2009*b*). *Acta Cryst.* E**65**, o3099–o3100. 10.1107/S1600536809047758 PMC2972131 21578827

[bb5] Hegde, J. C., Rai, G., Puranic, V. G. & Kalluraya, B. (2006). *Synth. Commun.* **36**, 1285–1290.

[bb6] Kalluraya, B. & Chimbalkar, R. M. (2001). *Indian J. Heterocycl. Chem.* **11**, 171–174.

[bb7] Kalluraya, B., Chimbalkar, R. M., Rai, G., Gururaja, R. & Shenoy, S. (2001). *J. Indian Council Chemists*, **18**, 1–5.

[bb8] Rai, N. S., Kalluraya, B., Lingappa, B., Shenoy, S. & Puranic, V. G. (2008). *Eur. J. Med. Chem.* **43**, 1715–1720. 10.1016/j.ejmech.2007.08.002 17923171

[bb9] Rathish, I. G., Kalim, J., Shamim, A., Sameena, B., Alam, M. S., Pillai, K. K., Surender, S. & Bagchi, V. (2009). *Bioorg. Med. Chem. Lett.* **19**, 255–258. 10.1016/j.bmcl.2008.10.105 19010670

[bb10] Sheldrick, G. M. (2008). *Acta Cryst.* A**64**, 112–122. 10.1107/S0108767307043930 18156677

[bb11] Spek, A. L. (2009). *Acta Cryst.* D**65**, 148–155. 10.1107/S090744490804362X PMC2631630 19171970

[bb12] Tawab, S. A., Mustafa, A. & Kira, M. (1960). *Nature (London)*, **186**, 165–166. 10.1038/186165a0 13837090

